# 2193. Vancomycin AUC:MIC Monitoring: Transitioning Safely from Inpatient to Outpatient Monitoring via OPAT

**DOI:** 10.1093/ofid/ofad500.1815

**Published:** 2023-11-27

**Authors:** Maureen Campion, Richard Aprile, Samantha Troy, Yuran Tsuchida, Tine Vindenes

**Affiliations:** Tufts Medical Center, Boston, Massachusetts; Tufts Medical Center, Boston, Massachusetts; Tufts Medical Center, Boston, Massachusetts; Tufts Medical Center, Boston, Massachusetts; Tufts Medical Center, Boston, Massachusetts

## Abstract

**Background:**

Due to the increased availability of AUC:MIC calculators and evidence showing limited benefit to troughs greater than 15 mcg/mL, the 2020 IDSA/ASHP consensus vancomycin therapeutic guideline recommends AUC:MIC monitoring for patients with severe MRSA infections. At Tufts Medical Center (TMC) in Boston, MA, patients receive vancomycin dosed by AUC:MIC for severe MRSA infections or requiring greater than two weeks of therapy. There is limited information on transitioning these patients to the outpatient setting. Obtaining two appropriately timed levels in the rehabilitation, home infusion or nursing home setting can be extremely challenging. Therefore, at TMC, a correlating trough range associated with an AUC:MIC of 400-600 was calculated based upon patient specific parameters. This trough range was communicated to the outpatient parenteral antimicrobial therapy (OPAT) team for monitoring outpatient, reducing the need to draw 2 levels within the same dosing interval. The purpose of this study was to identify issues associated with transitions of care with vancomycin AUC:MIC monitoring and to identify potential solutions.

**Methods:**

This was a retrospective review of patients receiving vancomycin dosed by AUC:MIC from September 2021 through March 2022. Patients who continued vancomycin with AUC:MIC monitoring on discharge were evaluated for challenges associated with their therapy by the OPAT coordinator. Data was collected on comorbidities, readmission to the hospital within 90 days, levels drawn incorrectly, patient non-compliance, need for re-education, and adjustment of therapy based on cultures.

**Results:**

Seventy-two (72) patients received vancomycin monitoring by AUC:MIC at TMC. Of these patients, forty-two (42) were discharged with OPAT services. Twenty-six (26) of patients received intravenous vancomycin and sixteen (16) were prescribed other antibiotics. Twenty (20) patients planned for at least four weeks of outpatient therapy. The results of the outcomes of patients on therapy are listed in Table 1.

**Figure:**
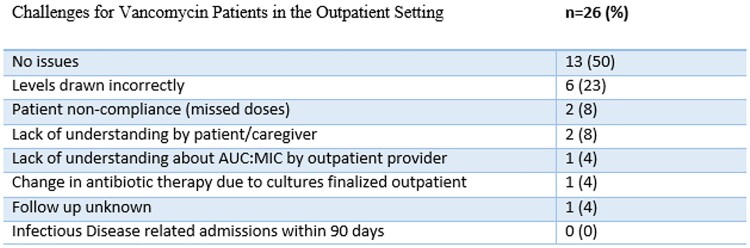

**Conclusion:**

Our small study demonstrates AUC:MIC monitoring can be done safely and effectively in the outpatient setting. An emphasis should be placed on the education of the outpatient providers.

**Disclosures:**

**Maureen Campion, PharmD**, shinoigi: Speaker

